# A Colorimetric Aptasensor for Rapid Detection of Sulfadimethoxine in Aquaculture

**DOI:** 10.3390/bios16070389

**Published:** 2026-07-18

**Authors:** Hong Liang, Jiahao Tan, Tingyu Wang, Yaomei Wang, Chen Zhang

**Affiliations:** 1 School of Geography and Oceanography, College of Materials and Chemistry Engineering, Minjiang University, Fuzhou 350108, China; lianghong@mju.edu.cn (H.L.);; 2 Fujian Key Laboratory of Conservation and Sustainable Utilization of Marine Biodiversity, Fuzhou Institute of Oceanography, Fuzhou 350108, China; 3 School of Pharmaceutical Sciences, Jilin University, Changchun 130021, China

**Keywords:** colorimetric sensor, aptamer, Sulfadimethoxine, aquaculture, G-quadruplex/hemin

## Abstract

Sulfadimethoxine (SDM) is a sulfonamide antibiotic widely used in the aquaculture of aquatic organisms. Its excessive residues in animal-derived food products can cause irreversible harm to human health and the environment. Current primary detection methods for SDM, such as instrumental methods and Immunoassay techniques, demonstrate high sensitivity and accuracy. However, their industrial application is impeded by laborious sample pretreatment, reliance on specific equipment, and dependence on specially trained personnel. Therefore, there is an urgent need to develop a simple and rapid method for detecting SDM residues. In this study, we constructed a novel colorimetric sensing platform based on functional nucleic acids for SDM detection. This sensor incorporates a nucleic acid aptamer capable of specifically recognizing SDM, a G-quadruplex/Hemin complex with peroxidase-like catalytic activity, and a shielding sequence that suppresses catalytic activity while undergoing SDM-induced conformational changes. The colorimetric signal was generated using a 3,3′,5,5′-Tetramethylbenzidine (TMB) chromogenic substrate, and the sensor’s performance was evaluated via absorbance measurements with a microplate reader. After optimizing detection conditions, the sensor exhibited a linear response to SDM concentrations ranging from 0.155 to 3.10 ng/mL, with a detection limit of 0.0796 ng/mL. Furthermore, the sensor demonstrated excellent selectivity and achieved recoveries of 83.0% to 107% in spiked aquaculture water and fish samples, with coefficients of variation below 10.4%, confirming its superior practicality for real-world sample analysis.

## 1. Introduction

China is the world’s largest aquaculture producer. With the continuous expansion of the aquaculture industry, environmental problems associated with aquaculture have become increasingly severe, leading to a high incidence of disease in aquatic animals [1]. Antibiotics play crucial roles in the prevention and treatment of bacterial diseases in aquaculture owing to their outstanding antibacterial properties [2,3]. Among them, sulfonamides (SAs) are one of the commonly used classes of antibiotics in aquaculture, offering advantages such as broad applicability [4], low cost, and stable chemical properties. They effectively inhibit Gram-negative/positive bacterial strains, including *Escherichia coli* and *Staphylococcus* spp., while also exhibiting significant antifungal activity against certain fungal groups. However, only 20–30% of the SAs administered in aquaculture are effectively absorbed and utilized by farmed organisms, whereas as much as 70–80% of the drugs ultimately remain in the aquaculture environment in undegraded forms [5,6]. These antibiotic residues can be transferred to humans through the food chain via bioaccumulation, causing toxic effects such as damage to the urinary and hematopoietic systems and allergic reactions [7]. Long-term low-dose exposure is also significantly associated with chronic toxicity [8]. Additionally, sulfonamides enter the environment through aquaculture wastewater discharge, inhibiting plant growth and development and reducing the activity of environmental microorganisms. Over time, this may even induce horizontal transfer of antimicrobial resistance genes [9]. Therefore, detecting SA residues in aquaculture products is of significant importance for environmental monitoring and food safety regulation.

In recent years, the most commonly used detection techniques for antibiotic residues in food are primarily instrumental analysis and immunoassay methods [10]. Among instrumental analysis methods, high-performance liquid chromatography (HPLC) coupled with mass spectrometry (MS) has emerged as the mainstream detection approach [11]. It allows for the precise quantification of target compounds and effectively minimizes analytical errors due to its high specificity and ultra-sensitivity [12]. Nevertheless, this method demands a high level of technical proficiency from operators. Moreover, it encounters challenges like high equipment costs and time-consuming sample pretreatment, which make it difficult to satisfy the requirements of rapid on-site antibiotic detection. In immunoassay methods, enzyme-linked immunosorbent assay (ELISA) is a widely used technique. ELISA leverages the specific reactions between antigens and antibodies to detect target compounds via enzyme-catalyzed colorimetric reactions [13]. Its sensitivity and accuracy are comparable to those of instrumental analysis methods [14]. However, its detection ability is reliant on antibodies, and it has drawbacks such as high cost, substantial batch-to-batch variation, strict storage requirements, and poor thermal stability [15]. Therefore, the development of a novel detection strategy that requires no large-scale instruments, features simple operation, rapid response, and cost control has become a research focus in the field of analytical chemistry [16].

Nucleic acid aptamers are single-stranded nucleic acid molecules selected via the Systematic Evolution of Ligands by Exponential Enrichment (SELEX) technique [17]. They bind target molecules with high affinity and specificity, and are often referred to as “chemical antibodies” [18]. Compared with antibodies, nucleic acid aptamers offer several advantages, including shorter preparation times, a broader target range, higher stability, lower cost, and greater ease of modification [19], making them well-suited as recognition elements for sensor development [20]. Among various sensors, colorimetric sensors have garnered considerable attention due to their unique advantages [21]. These sensors convert target recognition events into visible color changes, enabling direct visual observation or accurate quantification using UV-visible spectrophotometers, thereby greatly simplifying detection processes and eliminating reliance on large-scale instruments, making them highly suitable for rapid field testing [22].

Although functional nucleic acid-based colorimetric sensors have achieved substantial progress in detecting heavy metal ions and biological toxins, similar studies targeting sulfonamide antibiotics remain relatively scarce and commonly face challenges such as insufficient detection sensitivity and severe interference from real-world sample matrices. Herein, we utilize Sulfadimethoxine (SDM), a prevalent antibiotic within the sulfonamide antibiotic group, as a model to develop a colorimetric sensor founded on functional nucleic acids for the visual and rapid detection of SDM in aquaculture. The sensor comprises an ApS-G probe, a blocking (b) probe, hemin, 3,3′,5,5′-Tetramethylbenzidine (TMB) chromogenic solution, and a stopping solution. The ApS-G probe is composed of an aptamer targeting SDM [23] and a G-quadruplex [24]. The b probe could hybridize with the ApS-G probe to prevent G-quadruplex-hemin complex formation. In the presence of SDM, ApS-G binds to SDM, releasing the b probe, which subsequently induces the formation of a Hemin/G-quadruplex complex rich in guanine [25]. This complex exhibits peroxidase-like activity, catalyzing the oxidative chromogenic reaction of TMB, which results in a color change from colorless to blue (with enhanced absorption at 652 nm) and subsequently to yellow (with enhanced absorption at 450 nm) upon addition of stop solution. We optimized the assay conditions and evaluated its limit of detection (LOD) and sensitivity [26], and successfully applied it to detect SDM in real samples (aquaculture water and fish meat products). This colorimetric sensor provides a novel, rapid, and visual strategy for detecting sulfonamide antibiotics, with promising applications in environmental monitoring and food safety [27].

## 2. Materials and Methods

### 2.1. Chemicals and Reagents

Hemin was purchased from Aladdin Scientific, Shanghai, China. Sulfadimethoxine (SDM) was bought from Aladdin Scientific, Shanghai, China. Gentamicin (GEN) and Sulfamethoxazole (SMX) were obtained from Beijing Solarbio Science & Technology Co., Ltd., Beijing, China. Norfloxacin (OFX) was purchased from Shanghai Yuanye Bio-Technology Co., Ltd., Shanghai, China. Ultrasensitive TMB substrate solution and TMB stop solution were purchased from Beyotime Biotechnology, Shanghai, China. PBS buffer (10 mM phosphate-buffered saline, pH 7.4, containing 100 mM KCl) was purchased from Changde BKMAM Biotechnology Co., Ltd., Changde, Hunan, China. All reagents used were of analytical grade. PBS served as the washing buffer.

The APS-G, b1, b2, and b3 sequences were purchased from Sangon Biotech, Shanghai, China. The DNA sequences utilized in the research are detailed in Table 1.

### 2.2. Feasibility Analysis

First, mix 1.6 μL of 100 μM ApS-G with 2 μL of 100 μM b at 95 °C for 5 min, then slowly cool to room temperature over approximately 30 min to form the ApS-G + b complex. Add 0.5 μL of 800 μM SDM to the solution and incubate at room temperature with shaking for 1 h. Next, add 90 μL of TMB chromogenic reagent, followed by 1 μL of 80 μM hemin, and continue shaking at room temperature for 20 min. Transfer the mixture to a universal 96-well plate compatible with the ReadMax 300F microplate reader (Shanghai Shangpu Co., Ltd., Shanghai, China), add 100 μL of TMB stop solution, and measure the absorbance at 450 nm using the microplate reader. All experiments were performed at room temperature.

### 2.3. Control Experiments

To verify the signal origin, control experiments were performed under identical conditions. Three groups were tested. (1) “No Hemin” control: ApS-G (1.6 µL, 100 µM) and b2 (3.2 µL, 100 µM) were mixed, heated at 95 °C for 5 min, slowly cooled to room temperature over approximately 30 min, and then mixed with 90 µL TMB chromogenic reagent without hemin. (2) “b-probe mix” control: a mixture of b1, b2, and b3 (3.2 µL each, 100 µM) was incubated with 90 µL TMB and 1 µL hemin (80 µM) in the absence of ApS-G. (3) Positive control: ApS-G (1.6 µL, 100 µM) was incubated with 90 µL TMB and 1 µL hemin (80 µM) in the absence of b2. All groups were prepared in PBS buffer to a final volume of 100 µL. After incubation at room temperature for 20 min, 100 µL of stop solution was added, and absorbance at 450 nm was recorded.

To verify that the signal recovery is specifically triggered by SDM recognition of ApS-G, we designed a scrambled aptamer (Scr-ApS-G) as a negative control, which retains the same G-quadruplex domain and GC content as ApS-G but with a randomized SDM-recognition region. Six groups were tested: ApS-G, ApS-G + b2, ApS-G + b2 + SDM, Scr-ApS-G, Scr-ApS-G + b2, and Scr-ApS-G + b2 + SDM. For groups containing b2, ApS-G or Scr-ApS-G (1.6 µL, 100 µM) was first mixed with b2 (3.2 µL, 100 µM), heated at 95 °C for 5 min, and slowly cooled to room temperature over approximately 30 min to allow complex formation. Then, SDM (0.5 µL, 800 µM) was added to the corresponding groups and incubated at room temperature for 1 h. After 90 µL of TMB and 1 µL of hemin (80 µM) were added, the mixtures were incubated at room temperature for 20 min. All groups were prepared in PBS buffer with a final volume of 100 µL. Finally, 100 µL of TMB stop solution was added, and the absorbance at 450 nm was recorded.

### 2.4. Optimization of Reaction Parameters

The optimization of the sequence design: Specific steps for b-chain optimization: Mix the b1, b2, or b3 chains with ApS-G at 95 °C for 5 min, then slowly cool the mixture to room temperature. Subsequently, sequentially add 90 μL of TMB chromogenic solution and 1 μL of 80 μM hemin, followed by shaking at room temperature for 20 min. Finally, add 100 μL of TMB stop solution and measure the absorbance at 450 nm using a microplate reader.

The optimization of hemin concentration: Prepare 1.6 μL of 100 μM ApS-G and 2 μL of 100 μM b2, incubate at 95 °C for 5 min, then slowly cool to room temperature to form the ApS-G + b complex. Add 0.5 μL of 800 μM SDM and incubate at room temperature for 1 h. Subsequently, add 1 μL of hemin solution at different concentrations (final concentrations: 0.1 μM, 0.2 μM, 0.3 μM, 0.4 μM), followed by 90 μL of TMB chromogenic solution. Then, shake the mixture at room temperature for 20 min. Add 100 μL of TMB stop solution and measure absorbance at 450 nm using a microplate reader.

The optimization of the molar ratio between ApS-G and b probe: The molar ratios of ApS-G to b were set at 2:1, 1:1, 1:2, and 1:3. The reaction was carried out at 95 °C for 5 min, followed by slow cooling. Then, 0.5 μL of 800 μM SDM was added to the system and incubated at room temperature for 1 h. Subsequently, 1 μL of 80 μM Hemin and 90 μL of TMB chromogenic solution were sequentially added, and the mixture was shaken at room temperature for 20 min. Finally, 100 μL of TMB stop solution was added, and absorbance was measured at 450 nm using a microplate reader.

### 2.5. Sensitivity Evaluation

First, mix 1.6 μL of 100 μM ApS-G with 3.2 μL of 100 μM b at 95 °C for 5 min, then slowly cool to room temperature over approximately 30 min to form the ApS-G + b complex. Add varying concentrations of SDM (0, 0.031, 0.062, 0.155, 0.310, 0.621, 1.552, 3.103, 15.516, 31.033, 62.066, 155.165, 232.747, 310.33 ng/mL) to the above solution. After mixing, incubate at room temperature for 1 h. Then, add 90 μL of TMB chromogenic reagent and 1 μL of 80 μM hemin, and react under shaking at room temperature for 20 min. Subsequently, add 100 μL of TMB stop solution and measure the absorbance at 450 nm using a microplate reader. Finally, plot a calibration curve with SDM concentration on the *x*-axis and absorbance on the *y*-axis using GraphPad Prism 10.6.0 (890) (GraphPad Software, San Diego, CA, USA). Calculate the sensor’s limit of detection (LOD) based on the 3σ/S principle, where σ is the standard deviation of 10 independent blank measurements, and S is the slope of the calibration curve. The limit of quantification (LOQ) was calculated as LOQ = 10σ/S according to IUPAC recommendations.

### 2.6. Specificity Analysis

To assess the specificity of our aptasensor, SDM was tested along with several commonly used antibiotics in aquaculture, including gentamicin (GEN), sulfamethoxazole (SMX), and ofloxacin (OFX). The final concentrations of all interferents were 10 nM or 1000 nM. First, 1.6 μL of 100 μM ApS-G was mixed with 3.2 μL of 100 μM b, and the mixture was incubated at 95 °C for 5 min, followed by slow cooling to room temperature over approximately 30 min to form the ApS-G + b complex. Then, target SDM and interferents (at final concentrations of 10 nM and 100 nM, respectively) were added to the system in 0.5 μL aliquots each, thoroughly mixed, and incubated at room temperature for 1 h. Subsequently, 90 μL of TMB chromogenic reagent and 1 μL of 80 μM hemin were added, and the mixture was shaken at room temperature for 20 min. Finally, 100 μL of TMB stop solution was added. The sensor specificity was evaluated by measuring the absorption intensity at 450 nm in the presence of different interferents. All interferents were tested in triplicate, and the mean values from three independent experiments were plotted.

### 2.7. Real Sample Detection

Two types of real samples were used for spiked recovery experiments: aquaculture water and salmon meat powder. Salmon meat powder was purchased from a local supermarket. Aquaculture water was collected from a laboratory fish tank. For the salmon meat powder, a matrix extraction was performed prior to the spiking experiment: 5 mg of the powder was dispersed in 500 µL of ultrapure water and centrifuged at 12,000 rpm for 15 min. Then, the supernatant was collected for use. For the detection, an 8.5 µL reaction mixture was first prepared in a centrifuge tube, containing 3.7 µL of the salmon meat powder supernatant (or aquaculture water), 1.6 μL of 100 μM ApS-G aptamer solution, and 3.2 μL of 100 μM b2 sequence; these components were added sequentially and mixed well. Then, 0.5 μL of SDM solutions at different concentrations were added to achieve final concentrations of 0.5, 1, and 5 nM in the reaction system (equivalent to 0.155, 0.310, and 1.55 ng/mL, respectively). After reaction at room temperature for 1 h, 90 μL of TMB chromogenic solution and 1 μL of 80 μM Hemin were added, and the mixture was incubated for 20 min. Finally, 100 μL of TMB stop solution was added, and the absorbance at 450 nm was measured using a microplate reader. The recovery rate was calculated.

## 3. Results

### 3.1. Detection Principle and Feasibility Analysis of SDM

**Figure 1:**
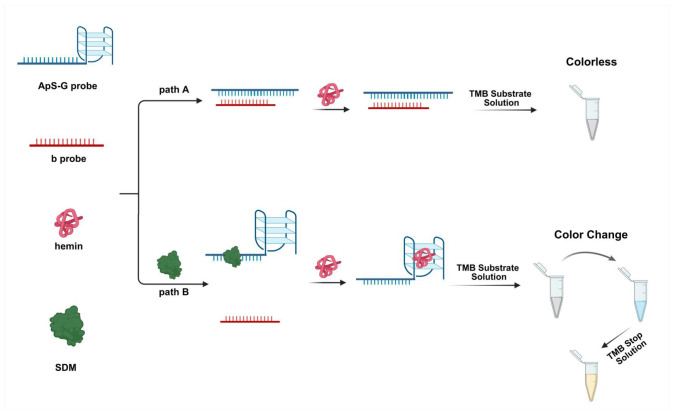
Schematic diagram of the principle of nucleic acid aptamer colorimetric sensor for Sulfadimethoxine (SDM) detection. (Created in BioRender. Liang, V. Available Online: https://BioRender.com/smzgva5 (accessed on 1 June 2026)).

Based on the specific recognition and binding between aptamer probes and their targets, as well as the peroxidase-like activity of G-quadruplexes/hemin complexes, we designed a nucleic acid aptamer-based colorimetric sensor for SDM detection. The working principle of the sensor is illustrated in Figure 1. The ApS-G + b complex consists of two probes: an ApS-G probe, which contains a nucleic acid aptamer that specifically recognizes SDM and a G-quadruplex sequence capable of binding hemin and exhibiting peroxidase activity; and a blocking sequence (b probe) that inhibits the catalytic activity of ApS-G. In the absence of the target SDM in the sample, ApS-G remains stably bound to the b chain, preventing its interaction with free hemin and thereby blocking the chromogenic reaction. Conversely, when SDM is present in the detection system, the target binds to ApS-G, causing the dissociation of the b chain from ApS-G. This restores the catalytic activity of ApS-G, enabling it to bind hemin and catalyze the oxidation of TMB, thus triggering a colorimetric response.

**Figure 2:**
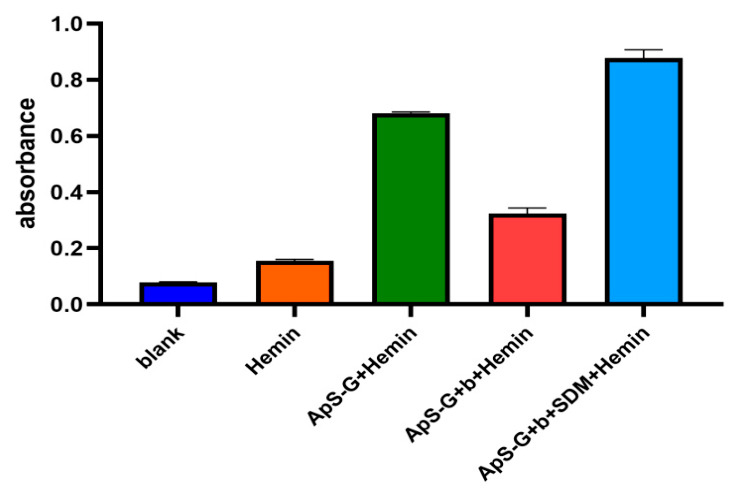
Feasibility verification of the functional nucleic acid-based colorimetric sensor for Sulfadimethoxine (SDM) detection.

First, we evaluated whether the designed colorimetric sensor could be used to detect SDM. As shown in Figure 2, the standalone ApS-G probe exhibited high absorbance. Upon addition of the b probe, its absorbance significantly decreased, indicating that the b probe binds to ApS-G and effectively inhibits its catalytic activity. When the target SDM was introduced into the reaction system containing the ApS-G + b complex, the solution’s absorbance markedly increased. This suggests that the SDM competes with the b probe for binding to ApS-G, thereby restoring its catalytic activity. This result preliminarily confirms that the designed colorimetric sensor is applicable for SDM detection.

Furthermore, to confirm that the observed colorimetric response strictly originates from the Hemin/G-quadruplex complex rather than from residual peroxidase activity of the b probe or impurities, control experiments were performed. As shown in Figure S1, the “No Hemin” control and the “b-probe mix” control (without ApS-G) both exhibited negligible absorbance, while the positive control (ApS-G + Hemin + TMB, without b2) displayed a strong signal. This result confirms that the colorimetric response is strictly dependent on the formation of the hemin/G-quadruplex complex.

Having confirmed the signal origin, we next designed a scrambled aptamer (Scr-ApS-G) to verify that the “OFF–ON” response is specifically mediated by SDM-induced b2 displacement. Scr-ApS-G retains the same G-quadruplex domain and GC content as ApS-G but with a randomized SDM-recognition region. As shown in Figure S2, Scr ApS-G alone (group 4) exhibited strong absorbance, comparable to that of ApS-G (group 1), confirming that the scrambled sequence retains the G-quadruplex structure and catalytic activity. Upon addition of b2, the signal of both systems was effectively suppressed (groups 2 and 5). When SDM was introduced, only the ApS-G system showed significant signal recovery (group 3), while the Sc ApS-G system remained inhibited (group 6). These results confirm that the OFF–ON response is specifically mediated by SDM-induced b2 displacement.

### 3.2. Condition Optimization

**Figure 3:**
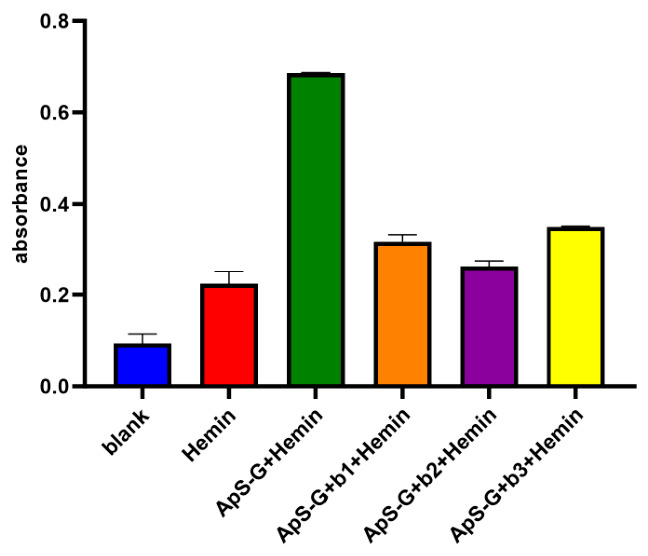
Effect of three different b chains (b1, b2, b3) on the catalytic activity of the sensor. Error bars represent the standard deviation of three parallel experiments.

The length of the complementary region between the b-probe and ApS-G probe, as well as their binding site, are critical factors influencing detection performance. If the complementary sequence is too short, the b probe fails to bind stably to ApS-G, resulting in incomplete blockage of the G-quadruplex sequence and leading to an abnormally high background signal. Conversely, if the complementary sequences are too long, the b probe binds too tightly to ApS-G, hindering the displacement efficiency of the target SDM and reducing detection sensitivity. Therefore, we optimized the b probe sequence design. As shown in Figure 3, among the three designed b probes (b1, b2, and b3), the lowest absorbance was observed when ApS-G was bound to b2, indicating that b2 exhibited the strongest inhibitory effect on the catalytic activity of ApS-G. Consequently, b2 was selected as the optimal b probe for subsequent experiments.

**Figure 4:**
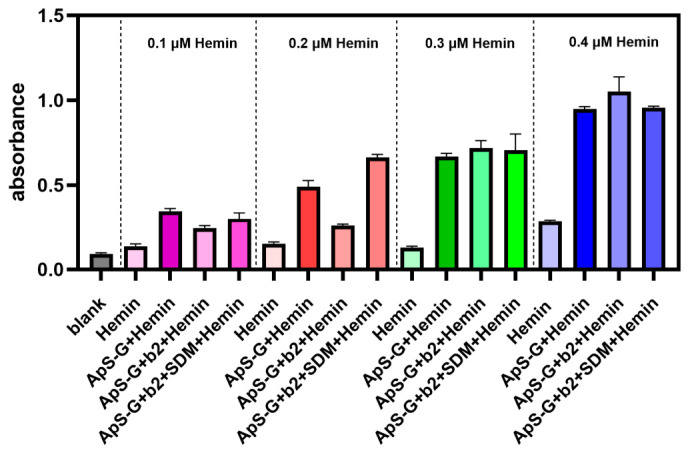
Optimization of hemin concentration. Error bars represent the standard deviation of three parallel experiments.

Hemin, as the core cofactor in G-quadruplex-catalyzed colorimetric reactions, directly influences the signal output of the catalytic system through its concentration. Optimal hemin concentration ensures efficient catalytic activity, whereas excessive hemin concentrations may induce catalytic activity even in the absence of G-quadruplex structures, leading to nonspecific background signals. Conversely, insufficient hemin concentrations impair proper G-quadruplex/hemin complex formation during detection, resulting in weakened signals and reduced sensitivity. To achieve optimal detection performance, we investigated the effects of varying hemin concentrations (0.1–0.4 μmol/L) on the absorbance of the sensing system. As shown in Figure 4, the absorbance values increased progressively with increasing hemin concentrations in both the ApS-G group and the ApS-G + b2 complex after addition of SDM. At a hemin concentration of 0.2 μM, the absorbance of the ApS-G + b2 complex group decreased significantly compared with that of the ApS-G group; however, it recovered upon addition of SDM, indicating that incorporation of the b2 probe effectively suppressed the catalytic activity of ApS-G, while SDM fully restored it. When concentrations exceeded 0.2 μM, no significant difference in absorbance was observed between the ApS-G and ApS-G + b2 complex groups, suggesting that high hemin concentrations generated strong background signals and that the b2 probe failed to inhibit ApS-G catalytic activity under these conditions. Therefore, we selected 0.2 μM as the optimal hemin concentration for the assay.

**Figure 5:**
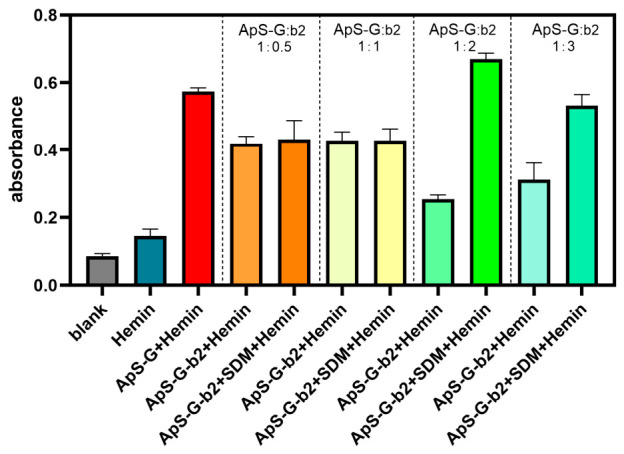
Optimization of the molar ratio of ApS-G to b2. Error bars represent the standard deviation of three parallel experiments.

The ratio of the ApS-G probe to b-probe directly affects the regulatory efficiency of the “on–off–on” system. If the b-probe concentration is too low, it fails to adequately suppress ApS-G’s catalytic activity, resulting in elevated background signals; conversely, excessive b-probe concentration prevents competitive displacement of target SDM from b-probe binding, leading to insufficient signal recovery. Therefore, we optimized the molar ratio of ApS-G to b probe (1:0.5, 1:1, 1:2, 1:3). As shown in Figure 5, when the ApS-G: b2 molar ratio was 1:0.5 or 1:1, the absorbance of the ApS-G + b2 complex exhibited a slight decrease compared to ApS-G alone, with no significant recovery after SDM addition. This may be attributed to b2 inhibiting only partial ApS-G’s catalytic activity, while uninhibited ApS-G binds more readily to SDM, preventing b2 dissociation and thus failing to restore catalytic activity. At an ApS-G: b2 molar ratio of 1:2, the absorbance of the ApS-G + b2 complex showed a marked decline compared to ApS-G alone, and SDM-induced signal recovery was most pronounced, achieving the maximum signal-to-noise ratio (ΔA = 0.414813). Further increasing the b2 concentration to a 1:3 ratio yielded no significant difference in the ApS-G-b2 absorbance compared to the 1:2, whereas SDM-mediated signal recovery was suppressed, resulting in a reduced overall signal-to-noise ratio (ΔA = 0.220). Consequently, we selected an optimal ApS-G: b molar ratio of 1:2 to ensure both low background signals and high response sensitivity in the system.

### 3.3. Specificity Evaluation

**Figure 6:**
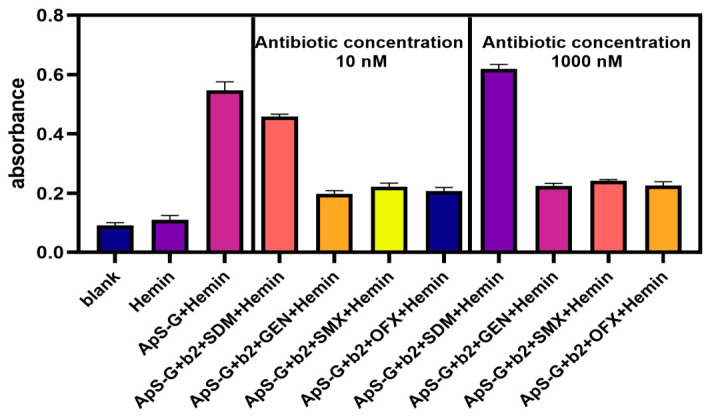
Compares the absorbance response of the sensing system in the presence of Sulfadimethoxine (SDM) and interferents (gentamicin (GEN), sulfamethoxazole (SMX), ofloxacin (OFX)). Error bars represent the standard deviation of three parallel experiments (*n* = 3).

To assess the specific recognition capability of the constructed sensor for SDM, we selected several commonly used antibiotics in aquaculture as interferents, including gentamicin (GEN), sulfamethoxazole (SMX) (a SDM structural analog), and ofloxacin (OFX), and conducted comparative tests under identical experimental conditions. The concentrations of each interferent were set at 10 nM and 1000 nM, respectively, matching those of SDM. The experimental results are shown in Figure 6. Only in the presence of SDM did the solution’s absorbance increase significantly, accompanied by noticeable color changes. In contrast, when the other antibiotics were present, the absorbance showed no significant change relative to the blank control and remained consistently and significantly lower than that observed with SDM. These results demonstrate that the sensor exhibits excellent specificity for SDM, primarily attributed to the high specificity of the aptamer toward the target.

### 3.4. Analytical Performance Study

**Figure 7:**
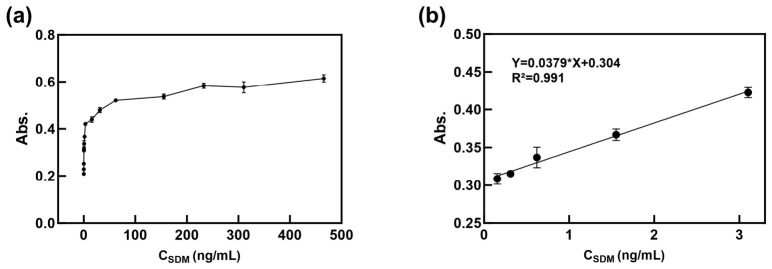
Sensitivity analysis of the colorimetric sensor for Sulfadimethoxine (SDM). (**a**) Absorbance spectra of the sensing system in the presence of different SDM concentrations (0~310.33 ng/mL); (**b**) linear fitting curve of absorbance changes versus SDM concentration. Error bars represent standard deviations from three parallel experiments (*n* = 3).

Under optimal experimental conditions, we evaluated the response capability of the constructed colorimetric sensor toward different concentrations of SDM to assess its detection sensitivity. As shown in Figure 7, the absorbance gradually increased with increasing SDM concentration, indicating that higher SDM concentration competitively displaced more b probe, thereby promoting greater formation of hemin/G-quadruplex and enhancing the catalytic chromogenic reaction. Linear regression of absorbance (Y) values versus SDM concentration (X) revealed a good linear relationship over the concentration range of 0.155–3.10 ng/mL, with the equation Y = 0.0379X + 0.304 and R^2^ = 0.991. According to the method recommended by the International Union of Pure and Applied Chemistry (IUPAC), the limit of detection (LOD) was calculated as LOD = 3σ/S (where σ is the standard deviation of 10 independent blank measurements and S is the slope of the calibration curve), yielding an LOD of 0.0796 ng/mL for SDM. The limit of quantification (LOQ), calculated as LOQ = 10σ/S, was determined to be 0.265 ng/mL. The LOD of our constructed sensor is comparable to those reported in the literature (summarized in Table 2). Unlike conventional sulfonamide antibiotic detection methods, which require antibodies, natural enzymes, or complex labeling, this sensor achieves superior sensitivity with a LOD, demonstrating significant advantages in detection performance.

### 3.5. Sample Spiking Recovery Rate

To evaluate the constructed colorimetric sensor’s applicability in complex real matrices, we selected representative actual samples (aquaculture water/salmon meat powder) and validated using the standard addition (spiked recovery) approach. Simple filtration was initially applied to the samples, followed by spiking with SDM standard solutions at three concentration levels: 0.155, 0.310, and 1.55 ng/mL. Absorbance measurements were performed under optimized conditions for each spiked sample, and the corresponding SDM concentrations were calculated from the calibration curves. The recovery rates and relative standard deviations (RSD, *n* = 3) were then determined (Table 3).

## 4. Discussion

This study developed a functional nucleic acid-based SDM colorimetric sensor that utilizes an aptamer competitive displacement strategy to regulate the catalytic activity of the hemin/G-quadruplex DNAzyme, enabling rapid and visual detection of SDM. Feasibility experiments validated the efficacy of the “off–on” sensing mechanism: In the absence of SDM, the b probe binds to the ApS-G probe via Watson–Crick base pairing, preventing formation of the hemin-G-tetramer complex and thereby inhibiting its catalytic activity, which blocks the chromogenic reaction. In the presence of SDM, SDM specifically binds to ApS-G, dissociating the b probe, releasing ApS-G, and enabling its folding into the active G-quadruplex structure; this restores hemin-dependent peroxidase-mimicking activity and catalyzes the oxidation of TMB to yield a blue color.

Optimization results demonstrated that the b-probe’s sequence directly influences background suppression in the absence of SDM, with the b2 probe exhibiting optimal inhibitory effects due to its formation of the most stable double-stranded structure with ApS-G. Further optimization of hemin concentration and ApS-G: b2 ratio revealed the critical role of competitive equilibrium in the system. The final optimized parameters (hemin concentration: 0.2 μmol/L; ApS-G: b2 ratio: 1:2) achieved an optimal balance between background suppression and signal recovery. Notably, when hemin concentration exceeded 0.2 µM, the inhibitory efficiency of b2 decreased markedly. The elevated background at higher hemin concentrations is likely attributable to the intrinsic peroxidase-like activity of free hemin, which can directly catalyze the oxidation of TMB even in the absence of G-quadruplex binding. All experiments were therefore conducted at 0.2 µM hemin, where the nonspecific contribution from free hemin is negligible [33,34].

Under optimal conditions, the detection limit of this sensor for SDM is 0.0796 ng/mL, with a linear range of 0.155–3.10 ng/mL. This label-free and antibody-free colorimetric sensor offers simple operation, low cost, and naked-eye visual detection capability. The LOD is comparable to or better than most existing sensors, though it remains less sensitive than certain electrochemical or fluorescent approaches (Table 2). Additionally, matrix interference from complex samples may affect signal stability, which was mitigated in this study by sample dilution. It is noteworthy that further improvements are still needed to enhance the sensor’s sensitivity, such as suppressing background signals and/or adopting more effective signal amplification strategies [35]. Specificity experiments confirmed excellent selectivity for SDM, with no significant response to non-target antibiotics such as gentamicin, sulfamethoxazole, or norfloxacin. Calibration recovery experiments using real samples (aquaculture water and salmon meat powder) yielded recovery rates ranging from 83.0% to 107.1%, with coefficients of variation below 10.4%. These results indicate high accuracy and precision in real sample analysis, minimal matrix interference, and suitability for rapid on-site detection. The innovation of this study lies in the following: For the first time, we constructed an SDM-based colorimetric sensor using an aptamer competitive displacement strategy to regulate the peroxidase-like activity of hemin/G-quadruplex DNAzymes. This approach enables a label-free, antibody-free, and enzyme-free sensing mode, thereby providing a novel technical solution for rapid, visual detection of sulfonamide antibiotics. Future research can further optimize this sensor, such as enhancing the affinity between aptamers and targets through chemical modification, improving its stability under external environmental conditions, integrating signal amplification strategies to increase sensitivity, and expanding its application to complex matrices such as fish meat and shrimp meat.

## 5. Conclusions

In this study, a functional nucleic acid-based colorimetric sensor for Sulfadimethoxine (SDM) was developed using an SDM aptamer as the recognition element and a G-quadruplex/hemin peroxidase-mimicking system coupled with an allosteric-responsive masking sequence as the signal transducer. After optimizing key parameters, the sensor achieved a detection limit of 0.0796 ng/mL and exhibited excellent selectivity for SDM. Spike recovery tests in aquaculture water (98.0–104.0%) and salmon meat powder (83.0–107%) confirmed good accuracy and anti-interference ability (CV < 10.4%). The method is antibody-free, enzyme-free, and label-free, enabling naked-eye visual detection, offering a simple and rapid on-site solution for sulfonamide antibiotic monitoring in aquaculture.

## Figures and Tables

**Table 1:** DNA sequences used in the experiment.

Name	Sequence (5′→3′)
ApS-G	GAGGGCAACGAGTGTTTATAGACTGGGAGGGAGGGAGGGA
Scr ApS-G	ACGTAGCAGGAGGGTTTATAGACTGGGAGGGAGGGAGGGA
b1	CTCCCAGTCTATAAAC
b2	CCCTCCCAGTCTATAAAC
b3	CCCAGTCTATAAAC

**Table 2:** Comparison of detection limits for SDM.

LOD	Signal Type	Source
0.11 ng/mL	Fluorescence detection	Liu, X.; et al. [28]
7.3 ng/mL	Fluorescence detection	Tang, J.; et al. [29]
0.76 ng/mL	Colorimetric detection	Yang, H.; et al. [30]
0.071 ng/mL	Colorimetric detection	Gao, X.; et al. [23]
0.02 ng/mL	Electrochemical detection	Du, M.; et al. [31]
0.21 ng/mL	Colorimetric detection	Tong, X.; et al. [32]

**Table 3:** Results of the Sulfadimethoxine (SDM) addition recovery experiment in actual samples (*n* = 3).

Sample	Concentration (ng/mL)	Recovery Concentration	Aptasensor Recovery Rate (%)	Coefficient of Variation
		(ng/mL)		(%)
Aquaculture water	0.155	0.158	102	9.48
	0.310	0.304	98.0	9.51
	1.55	1.61	104	4.45
Salmon meat powder	0.155	0.166	107	10.4
	0.310	0.346	111	7.00
	1.55	1.29	83.0	2.56

## Data Availability

The original contributions presented in this study are included in the article/Supplementary Materials. Further inquiries can be directed to the corresponding authors.

## Supplementary Materials

The following supporting information can be downloaded at: https://www.mdpi.com/article/10.3390/bios16070389/s1, Figure S1: Control experiments for signal origin validation. Figure S2: Mechanistic validation using scrambled aptamer (Scr-ApS-G).

## Abbreviations

The following abbreviations are used in this manuscript:

LOD	Limit of detection
LOQ	Limit of Quantification
SAs	sulfonamides
SDM	Sulfadimethoxine
HPLC	high-performance liquid chromatography
MS	mass spectrometry
SELEX	Systematic Evolution of Ligands by Exponential Enrichment
ELISA	enzyme-linked immunosorbent assay
IUPAC	International Union of Pure and Applied Chemistry
OFX	ofloxacin
GEN	gentamicin
