# Supplementary material for: A Colorimetric Aptasensor for Rapid Detection of Sulfadimethoxine in Aquaculture
Source: Biosensors (Basel). 2026 Jul 18;16(7):389. doi: 10.3390/bios16070389 (PMC13406975; doi:10.3390/bios16070389)
Supplement: Supplementary file 1 [file biosensors-16-00389-s001.zip › biosensors-4394561-supplementary.pdf]

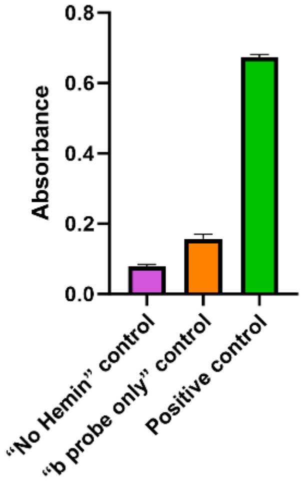

Figure S1. Control experiments for signal origin validation. Groups: (1) "No Hemin" control (ApS-G + b2 + TMB); (2) "b-probe only" control (b1 + b2 + b3 with Hemin + TMB); (3) positive control (ApS-G + Hemin + TMB). Error bars represent the standard deviation of three parallel experiments.

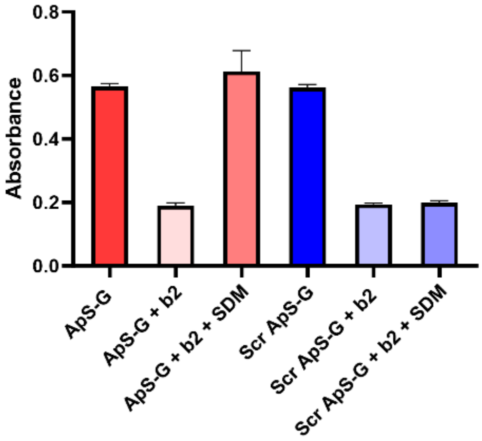

Figure S2. Mechanistic validation using a scrambled aptamer (Scr-ApS-G). Groups: (1) ApS-G; (2) ApS-G + b2; (3) ApS-G + b2 + SDM; (4) Scr-ApS-G; (5) Scr-ApS-G + b2; (6) Scr-ApS-G + b2 + SDM. Error bars represent the standard deviation of three parallel experiments.
